# Neuro-biological inferences into the depressive disorders of patients with chronic hepatitis C treated with interferon and ribavirin

**DOI:** 10.1186/1471-2334-14-S7-P7

**Published:** 2014-10-15

**Authors:** Dan Hurezeanu, Livia Dragonu, Daniela Ristea, Carmen Canciovici, Doina Ene, Maria Balan, Madalina Sandu, Dorel Cernea

**Affiliations:** 1 University of Medicine and Pharmacy Craiova, Romania; 2 “Victor Babeş” Clinical Hospital of Infectious Diseases and Pneumology, Craiova, Romania

## Background

This work is intended to establish a relation between two diseases, medical and psychiatric, both of them considered public health issues – chronic hepatitis C and depression. Interferon has been a great progress for the chronic hepatitis C patients’ therapy, but the treatment is encumbered by the occurrence of depression on 3-57% of patients. The purpose of this work is to assess the percent of the depressive disorder on chronic hepatitis C patients treated with interferon and ribavirin, as well as the monitoring of the related risk factors, upon depression occurrence and the evolution during anti-depressive treatment.

## Methods

The retrospective study has been performed during the period of January 2011-January 2014 on a group of 64 patients with chronic hepatitis C, receiving combined antiviral treatment (interferon and ribavirin), aged between 25-64 years, observing the criteria of inclusion into treatment. The moment of psychiatric examination request has been recorded, 3.1% needing psychiatric examination before starting the therapy, 28.1% having symptoms which occurred during treatment.

## Results

The psychiatric examination has revealed as speeding causes of the depressive disorder the loss of social support for 39.5%, the biologic syndrome for 23%, and the loss of family support for 15.4%, and the depressive history for only 5.5%. The percent of the depressive patients after the starting moment of the antiviral therapy has emphasized the occurrence of the depressive disorder on 83% of patients during the first 0-12 weeks. The maximum incidence of cases has been recorded on patients with ages between 20-45 years. The cases distribution has been favorable to patients from urban area -15 cases, versus 3 cases from rural area.

## Conclusion

The antiviral therapy for chronic hepatitis C has determined the occurrence of different levels of depression for 28.1% of the patients being studied. The anti-depressive treatment associated with the antiviral one has been efficient, decreasing the abandon rate of the antiviral therapy.

